# Performance indicators in women’s and men’s biathlon relay

**DOI:** 10.1186/s13102-025-01160-z

**Published:** 2025-04-28

**Authors:** Marko S. Laaksonen, Glenn Björklund

**Affiliations:** Swedish Winter Sports Research Centre, Department of Health Sciences, Mid Sweden University, Östersund, Sweden

**Keywords:** Competition, Cross-country skiing, Final rank, Rifle shooting, Tactics, Team performance

## Abstract

This study aimed to investigate how different legs as well as cross-country skiing and shooting performances, associate to final rankings in biathlon relay competitions for both women and men. Data including rank, finish/leg time (LT), course (CT), range (RT) and penalty (PT) times, as well as number of shots (NS) and penalty loops (NPL), were collected from the International Biathlon Union’s database over two seasons, comprising 12 competitions for all teams ranked 1–20. Teams were categorized as G3 (rank 1–3), G10 (rank 4–10) and G20 (rank 11–20). Kruskal-Wallis’ test was used to compare the variables between the groups in total for an entire relay competition, and for each leg. For women, LT was longer for G20 across all four legs due to longer CT, and for G10 during legs 2 and 4 due to longer RT compared to G3 (*p* < 0.05). For men, LT was longer for G20 during all legs due to longer CT and RT (legs 2–4), and for G10 during legs 3 and 4 due to longer CT compared to G3 (*p* < 0.05). The present results suggest therefore that the shooting performance for women (especially shorter RT) during legs 2 and 4, and skiing performance for men during legs 3 and 4, are most decisive for final performance during a biathlon relay.

## Introduction

Biathlon combines cross-country skiing (effort-based performance) and rifle marksmanship (skill-based performance) at both prone and standing positions [1]. Biathlon performance is therefore determined by several factors. Skiing speed, which refers to the time spent skiing the course, is one of those factors. Another is range time, which is the time spent on the shooting range area, i.e. from 10 m before the last shooting lane (#30) to 10 m after the first shooting lane (#1). Range time also includes the shooting time, i.e. the time spent on the shooting mat when shooting, which also has an impact on performance. Additionally, penalty time is a critical factor that refers to the time between the end of shooting range and first intermediate time spot directly after the penalty loop. The number of penalty loops is dependent on shooting accuracy, as explained below.

Numerous previous studies of biathlon have aimed to elucidate the magnitude of the aforementioned performance-related factors on final outcomes in different individual competitions formats. For instance, in individual competition it has been suggested that cross-country skiing and shooting contribute similarly [2], whereas in sprint the skiing speed is the most important factor for final performance [3]. However, in another study, shooting accuracy has been found to be the primary factor for overall performance in both disciplines [4]. In pursuit and mass start, shooting accuracy has been shown to have the strongest association with final rank [5, 6]. Despite the increasing body of biathlon research, the relay format remains sparsely studied, with limited understanding on how various performance indicators impact relay team performances.

In biathlon, a relay team comprise four biathletes representing the same nation and sex. The start of the relay (first leg) is performed as a simultaneous mass start, while the second, third and fourth legs resemble a pursuit start. Each leg consists of three skiing loops (2 km for women, 2.5 km for men) separated by one prone and one standing shooting occasion. Thus, the total skiing distance covered is 24 km and 30 km of skiing for women and men, respectively, and includes a total of four prone and four standing shooting occasions. In biathlon relay competitions, the rules differ compared to individual competitions as during each shooting occasion, the biathlete has three spare shots to use if she/he misses a target. If all three spare shots are used while there are still missed targets left, the biathlete must ski the same number of penalty loops (150 m each) as the number of missed targets [1] and therefore, the total skiing distance increases.

In biathlon relay events, not only is it challenging to select the best possible biathletes for the team defined by their performance characteristics, but also to assemble the order of the athletes within the team to optimize team performance [7, 8]. Consequently, skiing speed and shooting performance of all four biathlon team members must be considered, as the variation in these performance-determining factors between biathletes, and thereby in which order they should participate between the relay legs, may affect the outcome of the competition. For instance, at the beginning of the relay, most of the teams are likely skiing more or less together which enables weaker skiers to achieve help of drafting during the skiing part. During later stages of the relay, sprinting ability but also better shooting performance especially in standing shooting might be beneficial.

From another point of view, the number of biathletes allowed to start in sprint and individual competitions is also partly based on the Nation cup score set by the International Biathlon Union (IBU) competition rules. The Nation cup score is calculated from each nation’s results from sprint and individual competitions, as well as from different relay competitions [1]. Therefore, the relay competition also plays a crucial role in determining the number of biathletes each nation can enter in sprint and individual competitions. Accordingly, an understanding of the performance-determining factors in biathlon relay is warranted.

The present study, therefore, aimed to describe how different legs as well as cross-country skiing (course time, CT) and shooting performance (range time, RT; number of shots, NS; penalty time, PT; and number of penalty loops, NPL) associate to final rank in biathlon relay competitions for both women and men.

## Methods

### Procedure

All data were retrieved from openly available International Biathlon Union’s (IBU) public datacenter at http://biathlonresults.com [9], with permission from IBU. Data were collected for all teams ranked 1–20 in all IBU World Cup relay competitions for women and men during the seasons 2020–2021 and 2021–2022, consisting of a total of 12 competitions for both sexes. If a team was lapped during the competition (i.e. team gets final rank but not finish time), that team was excluded from the analysis. The dataset included rank, finish time, course (CT), range (RT), and penalty times (PT), as well as number of shots (NS) and penalty loops (NPL) for the entire team (*overall performance*), and for each leg (legs 1, 2, 3 and 4), including the leg time (LT) (*leg performance*). The data were then categorized into three groups in both women and men (G3, rank 1–3; G10, rank 4–10, and G20, rank 11–20) based on the final rank to be able to compare performance determining factors between different performance levels as previously reported [4, 5, 10]. This approach also enabled to have a comparable number of teams in each group.

### Data and statistical analyses

Normal distribution was first checked using the Kolmogorov Smirnov test. As most of the variables did not conform to normal distribution, a further analysis was performed using non-parametric methods, and is presented as median [IQR] or type values. Second, a Kruskal-Wallis test with epsilon squared (ε^2^) for determination of effect size was used to compare CT, RT, PT, NS, and NPL between G3, G10, and G20 in total for the entire relay competition, as well as for each leg. Epsilon squared were classified as follows: negligible (0.00 < 0.01), weak (0.01 < 0.04), moderate (0.04 < 0.16), relatively strong (0.16 < 0.36), strong (0.36 < 0.64), and very strong (0.64 < 1.00) [11]. Third, Kendall’s Tau (τ) was used to identify associations between rank for separate legs (1–3) and the final rank. The τ value was thereafter transformed to Pearson’s r values accordingly [12]. The transformed r values and the count of teams that successfully completed the relay were subsequently subjected to further analysis for each leg (1–3). Effect size values for Pearson’s *r* were categorized as follows: small (*r* = 0.1), medium (*r* = 0.3), and large (*r* ≥ 0.5) [13]. Further, Cohen’s d was calculated for each r value obtained from τ (d = 2r/[1-r2)0.5]). Effect size values for Cohen’s d were considered small (d = 0.2), medium (d = 0.5) and large (d ≥ 0.8) [14]. A Fisher’s z transformation was applied for z score comparisons [15] between women and men, and the strength of relationship for each leg and final rank. All statistical analyses were performed using *Jamovi* [16]. The α was set a priori of < 0.05.

## Results

In total, 36 and 84 separate teams were included in G3 and G10 in both women and men. For G20, due to lapped teams in some relays, the final dataset included 207 and 226 teams for women and men, respectively.

### Overall performance

The total finish time, CT, RT, and PT were longer for G20 compared to G3 for both women and men (Table 1). In addition, the finish time, CT and RT, as well as PT (men only) were longer for G20 when compared to G10. G10 also had a longer finish time and CT compared to G3. Both G10 and G20 exhibited higher NS and NPL (women only) than G3. NS and NPL (men only) were also higher for G20 compared to G10.

**Table 1:** Biathlon relay components for different performance groups

			G3	G10	G20	χ^2^ (df), *p*-value, effect size (ε^2^)
		**Women**				
Total finish time (min)			111:10 [3:30]	113:19 [4:13]*	115:43 [4:26]*$	χ^2^ (2) = 43.6, *p* < 0.001, ε^2^ = 0.215
CT (min)			62:41 [2:42]	63:54 [3:40]*	65:44 [4:07]*$	χ^2^ (2) = 29.1, *p* < 0.001, ε^2^ = 0.143
RT (min)			7:46 [1:11]	8:15 [1:11]	8:41 [1:22]*$	χ^2^ (2) = 18.3, *p* < 0.001, ε^2^ = 0.090
PT (min)			0:59 [0:26]	1:11 [0:39]	1:15 [0:53]*	χ^2^ (2) = 7.3, *p* = 0.026, ε^2^ = 0.036
NS (*n*)			48 [3.25]	50 [4]*	51 [4]*$	χ^2^ (2) = 22.9, *p* < 0.001, ε^2^ = 0.113
NPL (*n*)			0 [0.25]	1 [1]*	1 [2]*	χ^2^ (2) = 19.3, *p* < 0.001, ε^2^ = 0.095
		**Men**				
Total finish time (min)			114:26 [3:51]	116:40 [3:30]*	119:14 [4:22]*$	χ^2^ (2) = 58.3, *p* < 0.001, ε^2^ = 0.259
CT (min)			66:24 [2:40]	67:55 [2:36]*	69:45 [3:12]*$	χ^2^ (2) = 49.5, *p* < 0.001, ε^2^ = 0.243
RT (min)			7:30 [1:02]	7:38 [0:56]	8:18 [0:57]*$	χ^2^ (2) = 42.5, *p* < 0.001, ε^2^ = 0.190
PT (min)			1:00 [0:22]	1:03 [0:26]	1:14 [0:51]*$	χ^2^ (2) = 13.7, *p* < 0.001, ε^2^ = 0.061
NS (*n*)			47 [4]	49 [4]*	51 [4]*$	χ^2^ (2) = 49.0, *p* < 0.001, ε^2^ = 0.218
NPL (*n*)			0 [1]	0 [1]	1 [2]*$	χ^2^ (2) = 25.8, *p* < 0.001, ε^2^ = 0.115

*CT*, total course, *RT*, range time, *PT*, penalty time, *NS*, number of shots, *NPL*, number of penalty loops, *G3*, group of final rank 1–3, *G10*, group of final rank 4–10, *G20*, group of final rank 11–20. Data are presented as median [IQR]

* *p* < 0.05 from G3

$ *p* < 0.05 from G10

### Leg performance

**Fig. 1:**
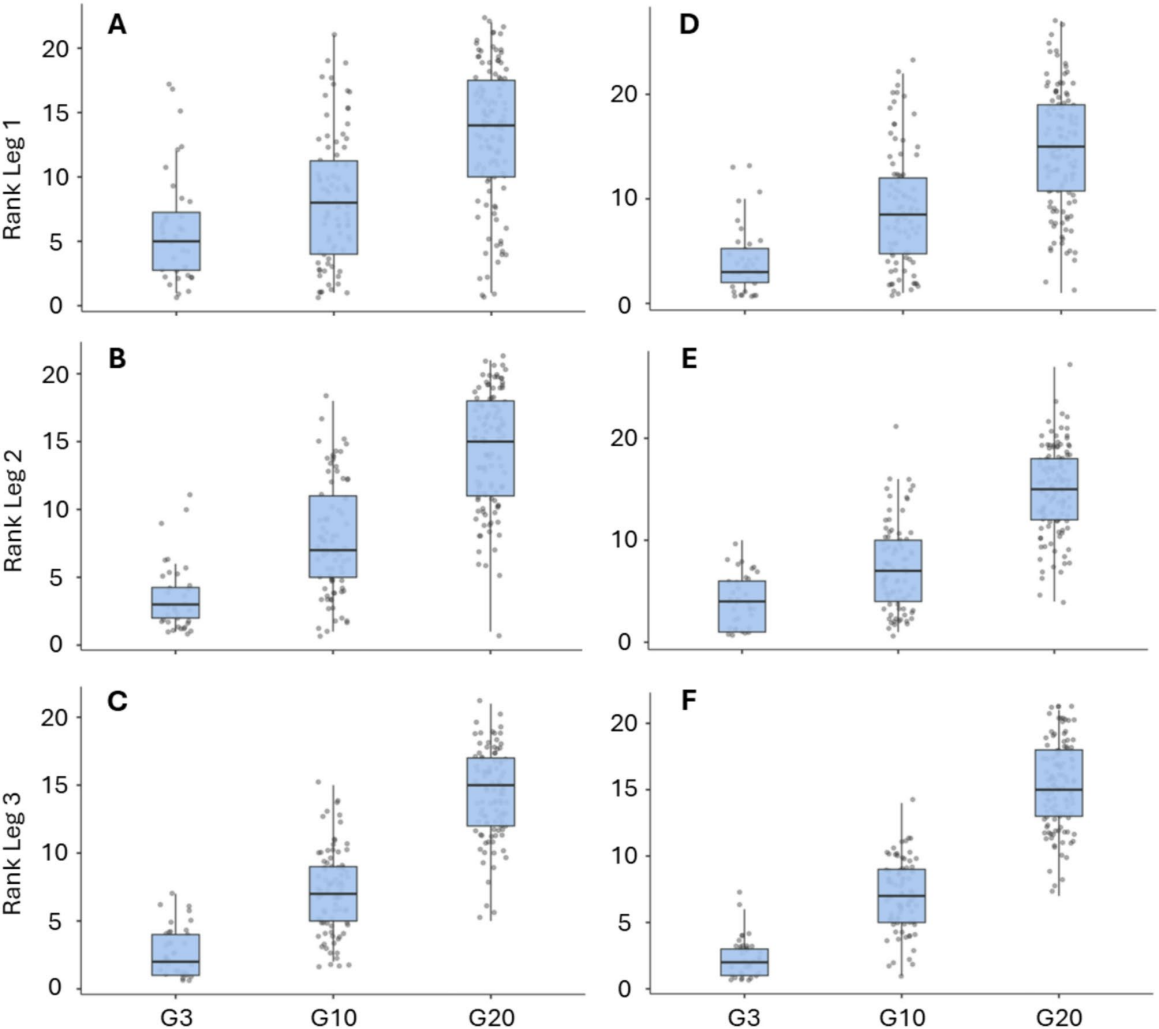
The ranks after legs 1, 2 and 3 for women (**A**-**C**) and men (**D**-**F**) in different performance groups (G3, rank 1–3; G10, rank 4–10; G20, rank 11–20) presented as a box plot including data points for all teams

The median rank for G3, G10 and G20 after legs 1, 2, and 3 for both women and men are presented in Fig. 1. The rank after legs 1, 2, and 3 were increasingly associated to final rank for both women (d = 1.4, d = 2.6, and d = 5.3, respectively) and men (d = 1.7, d = 2.7, and d = 5.9, respectively); see Table 2. No significant differences were observed between sexes in the strength of these relationships, as indicated by z-score statistics (Table 2).

**Table 2:** The association between leg rank and final rank in women and men relay

	Women		Men		Women vs. Men	
	r	*p*	r	*p*	z	*p*
Leg 1	0.57	< 0.01	0.64	< 0.01	1.15	0.25
Leg 2	0.80	< 0.01	0.81	< 0.01	0.27	0.79
Leg 3	0.94	< 0.01	0.95	< 0.01	0.96	0.34

*R*, Pearson correlation coefficient transformed from Kendall’s Tau (τ), *z*, z score according to Fisher’s z’ transformation

For women, LT was longer for G20 across all four legs and for G10 during legs 2 and 4 compared to G3 (Table 3). Additionally, G20 had a longer LT compared to G10 during legs 2, 3, and 4. The longer LT for G20 compared to G3 was due to longer CT (all legs), as well as longer RT (legs 2 and 4). The difference in LT between G10 and G3 was due to longer RT during legs 2 and 4, as well as longer CT during leg 3. NS was higher for G20 during legs 1, 2, and 4 and for G10 during leg 2 compared to G3. NPL was higher for G20 compared to G3 during legs 2 and 4. No difference in NS or NPL was observed between G20 and G10.

**Table 3:** Biathlon relay components for different performance groups for all four legs in women relay

		Leg 1	Leg 2	Leg 3	Leg 4
	G3	17.40 (0.54)	17.41 (0.53)	17.56 (1.13)	17.30 (0.58)
LT (min.sec)	G10	18.01 (1.05)	18.10 (1.10)*	18.18 (1.16)	18.03 (1.32)*
	G20	18.22 (1.14)*	18.50 (1.23)*$	19.06 (1.07)*$	19.07 (1.26)*$
		*χ2 (2) = 10.8*, *p* < 0.01,* ε2 = 0.053*	*χ2 (2) = 21.5*, *p* < 0.001,* ε2 = 0.106*	*χ2 (2) = 29.4*, *p* < 0.001,* ε2 = 0.145*	*χ2 (2) = 55.5*, *p* < 0.001,* ε2 = 0.273*
	G3	15.41 (0.57)	15.34 (0.56)	15.41 (1.04)	15.32 (049)
CT (min.sec)	G10	15.51 (0.46)	15.52 (0.59)	16.04 (1.01)*	15.49 (1.14)
	G20	16.13 (1.10)*	16.15 (1.19)*$	16.38 (1.17)*$	16.35 (1.39)*$
		*χ2 (2) = 9.7*, *p* < 0.01,* ε2 = 0.048*	*χ2 (2) = 12.6*, *p* < 0.01,* ε2 = 0.062*	*χ2 (2) = 25.7*, *p* < 0.001,* ε2 = 0.127*	*χ2 (2) = 402*, *p* < 0.001,* ε2 = 0.198*
	G3	1.51 (0.21)	1.53 (0.37)	2.08 (0.22)	1.52 (0.19)
RT (min.sec)	G10	1.53 (0.24)	2.07 (0.28)*	2.06 (0.32)	2.01 (0.23)*
	G20	2.02 (0.30)	2.14 (0.32)*	2.14 (0.33)	2.08 (0.30)*$
		*χ2 (2) = 3.8*, *p* = 0.148,* ε2 = 0.019*	*χ2 (2) = 16.1*, *p* < 0.001,* ε2 = 0.080*	*χ2 (2) = 1.5*, *p* = 0.474,* ε2 = 0.07*	*χ2 (2) = 18.8*, *p* < 0.001,* ε2 = 0.093*
	G3	0.14 (0.06)	0.14 (0.06)	0.15 (0.07)	0.14 (0.07)
PT (min.sec)	G10	0.14 (0.06)	0.15 (0.08)	0.14 (0.07)	0.15 (0.07)
	G20	0.13 (0.07)	0.15 (0.24)	0.15 (0.25)	0.14 (0.10)
		*χ2 (2) = 0.5*, *p* = 0.760,* ε2 = 0.003*	*χ2 (2) = 1.1*, *p* = 0.565,* ε2 = 0.006*	*χ2 (2) = 0.2*, *p* < 0.916,* ε2 = 0.000*	*χ2 (2) = 0.5*, *p* = 0.773,* ε2 = 0.003*
	G3	12 (1)	12 (2)	13 (2)	12 (1)
NS (n)	G10	12 (2)	13 (2)*	13 (2)	13 (2)
	G20	13 (3)*	13 (2)*	13 (2)	13 (3)*
		*χ2 (2) = 9.1*, *p* < 0.05,* ε2 = 0.044*	*χ2 (2) = 13.7*, *p* < 0.001,* ε2 = 0.067*	*χ2 (2) = 2.0*, *p* = 0.373,* ε2 = 0.001*	*χ2 (2) = 7.4*, *p* < 0.05,* ε2 = 0.037*
	G3	0 (0)	0 (0)	0 (0)	0 (0)
NPL (n)	G10	0 (0)	0 (0)	0 (0)	0 (0)
	G20	0 (0)	0 (1)*	0 (1)	0 (0)*
		*χ2 (2) = 0.3*, *p* = 851,* ε2 = 0.002*	*χ2 (2) = 7.0*, *p* < 0.05,* ε2 = 0.034*	*χ2 (2) = 6.4*, *p* < 0.05,* ε2 = 0.032*	*χ2 (2) = 7.5*, *p* < 0.05,* ε2 = 0.037*

*LT*, leg time, *CT*, total course, *RT*, range time, *PT*, penalty time, *NS*, number of shots, *NPL*, number of penalty loops, *G3*, group of final rank 1–3, *G10*, group of final rank 4–10, *G20*, group of final rank 11–20. Data are presented as median [IQR]

* *p* < 0.05 from G3

$ *p* < 0.05 from G10

For men, LT was longer for G20 during all legs and for G10 during legs 3 and 4 compared to G3 (Table 4). Moreover, G20 had a longer LT compared to G10 during all legs. For G20, the difference in LT compared to G3 was due to longer CT across all four legs but also from longer RT during legs 2, 3, and 4. Additionally, G20 had a longer PT compared to G3 during leg 3. The difference in LT between G10 and G3 was attributed to a longer CT during legs 3 and 4. NS was higher for G20 during legs 2, 3, and 4 and for G10 during legs 3 and 4 compared to G3. In addition, G20 had higher NS compared to G10 during legs 2 and 3. NPL was higher for G20 during leg 3 in comparison to G3 and G10.

**Table 4:** Biathlon relay components for different performance groups for all four legs in men relay

		Leg 1	Leg 2	Leg 3	Leg 4
	G3	18.52 (1.02)	18.53 (1.11)	18.49 (1.01)	18.48 (0.59)
LT (min.sec)	G10	19.12 (0.54)	19.03 (1.07)	19.29 (1.11)*	19.29 (1.10)*
	G20	19.41 (1.16)*$	19.45 (1.18)*$	20.25 (1.19)*$	20.14 (1.44)*$
		*χ2 (2) = 23.8*, *p* < 0.001,* ε2 = 0.106*	*χ2 (2) = 30.6*, *p* < 0.001,* ε2 = 0.136*	*χ2 (2) = 64.4*, *p* < 0.001,* ε2 = 0.286*	*χ2 (2) = 46.5*, *p* < 0.001,* ε2 = 0.206*
	G3	16.40 (0.46)	16.29 (0.45)	16.32 (0.45)	16.29 (0.41)
CT (min.sec)	G10	16.59 (0.49)	16.47 (0.48)	17.02 (0.59)*	16.56 (0.56)*
	G20	17.16 (1.02)*$	17.18 (0.52)*$	17.32 (1.04)*$	17.27 (1.13)*$
		*χ2 (2) = 25.1*, *p* < 0.001,* ε2 = 0.112*	*χ2 (2) = 29.2*, *p* < 0.001,* ε2 = 0.130*	*χ2 (2) = 54.5*, *p* < 0.001,* ε2 = 0.242*	*χ2 (2) = 46.5*, *p* < 0.001,* ε2 = 0.207*
	G3	1.50 (0.17)	1.53 (0.25)	1.49 (0.18)	1.51 (0.25)
RT (min.sec)	G10	1.48 (0.22)	1.48 (0.26)	2.01 (0.28)	1.54 (0.23)
	G20	1.52 (0.21)	2.02 (0.24)*$	2.15 (0.35)*$	2.10 (0.31)*$
		*χ2 (2) = 2.7*, *p* = 0.258,* ε2 = 0.012*	*χ2 (2) = 18.5*, *p* < 0.001,* ε2 = 0.082*	*χ2 (2) = 32.1*, *p* < 0.001,* ε2 = 0.143*	*χ2 (2) = 24.18*, *p* < 0.001,* ε2 = 0.108*
	G3	0.13 (0.06)	0.13 (0.07)	0.13 (0.06)	0.15 (0.06)
PT (min.sec)	G10	0.13 (0.05)	0.13 (0.06)	0.14 (0.06)	0.15 (0.07)
	G20	0.12 (0.07)	0.13 (0.06)	0.16 (0.24)$	0.14 (0.21)
		*χ2 (2) = 1.5*, *p* = 0.479,* ε2 = 0.007*	*χ2 (2) = 0.1*, *p* = 0.937,* ε2 = 0.000*	*χ2 (2) = 8.3*, *p* < 0.05,* ε2 = 0.037*	*χ2 (2) = 0.2*, *p* = 0.907,* ε2 = 0.000*
	G3	12 (1)	12 (1)	12 (1)	12 (2)
NS (n)	G10	12 (2)	12 (2)	13 (3)*	12 (2)*
	G20	12 (2)	13 (2)*$	13 (3)*$	13 (2)*
		*χ2 (2) = 0.4*, *p* = 0.815,* ε2 = 0.002*	*χ2 (2) = 15.3*, *p* < 0.001,* ε2 = 0.068*	*χ2 (2) = 26.6*, *p* < 0.001,* ε2 = 0.118*	*χ2 (2) = 14.0*, *p* < 0.001,* ε2 = 0.062*
	G3	0 (0)	0 (0)	0 (0)	0 (0)
NPL (n)	G10	0 (0)	0 (0)	0 (0)	0 (0)
	G20	0 (0)	0 (0)	0 (1)*$	0 (1)
		*χ2 (2) = 2.2*, *p* = 0.342,* ε2 = 0.010*	*χ2 (2) = 2.4*, *p* = 0.309,* ε2 = 0.010*	*χ2 (2) = 26.7*, *p* < 0.001,* ε2 = 0.119*	*χ2 (2) = 2.6*, *p* = 0.274,* ε2 = 0.012*

*LT*, leg time, *CT*, total course, *RT*, range time, *PT*, penalty time, *NS*, number of shots, *NPL*, number of penalty loops, *G3*, group of final rank 1–3, *G10*, group of final rank 4–10, *G20*, group of final rank 11–20. Data are presented as median [IQR]

* *p* < 0.05 from G3

$ *p* < 0.05 from G10

## Discussion

The aim of the present study was to investigate how leg performance as well as cross-country skiing (CT) and shooting performances (RT, PT, NS and NPL) are associated to final rankings in biathlon relay competition. The current data suggest first that the teams ranked 11–20 (G20; both women and men) have significantly longer LT, primarily due to longer CT across all four legs compared to G3. Second, legs 2 and 4 for women and legs 3 and 4 for men seem to be the most crucial legs distinguishing the groups ranked 1–3 (G3) and 4–10 (G10), and third, the impact of skiing speed (CT) and/or shooting performance (RT and NS) may play different roles in women’s versus men’s biathlon relay performance.

### Women relay

During leg 1, G3 and G10 had comparable LT because of similar CT and RT. Furthermore, there were no differences in PT, NS, or NPL between these groups during leg 1. Additionally, based on the median rank, it is evident that these first 10 teams were shuffled after leg 1; thus, G10 teams still had a chance to secure a podium finish, as demonstrated in Table 2; Fig. 1. However, during leg 2, G10 showed significantly longer RT, which led to an increased time gap against G3 and, further, during leg 3, G10 also had longer CT compared to G3. Finally, during leg 4, G10 again had longer RT compared to G3. Altogether, it is mostly the longer RT during legs 2 and 4 for G10 which distinguishes from G3. This highlights the importance of fast and accurate shooting, i.e. reduced number of extra shots, specifically during leg 2 when many teams are still skiing and shooting together, mimicking mass start and pursuit competitions. Indeed, one previous study has suggested that the presence of more co-acting biathletes at the shooting range during pursuit may be associated with faster shooting but not affect the shooting accuracy [17], and this could also be applicable for the relay.

### Men relay

During legs 1 and 2, G3 and G10 exhibited similar LT, CT, and RT. While the (statistically non-significant) difference in CT between these groups was rather large, G10 seemed to compensate for this difference with at least comparable RT values to G3, resulting in no overall difference in LT. Also, the median rank after leg 2 for G3 was 4, with relatively narrow range between the minimum and maximum ranks, indicating that teams in G3 and G10 were mixed at this stage, as shown in Fig. 1. However, during legs 3 and 4, G3 was skiing significantly faster than G10, thereby increasing the time gap against G10. Speculatively, teams having weaker skiers on legs 1 and 2 might have had less physical effort during skiing due to aerodynamic advantage of drafting and therefore, skiing performance discriminated G3 and G10 during legs 3 and 4. Indeed, the shooting accuracy was lower for G10 during legs 3 and 4, but this did not lead to longer RT, thereby emphasizing that the increased time gap to G3 was mostly due to longer CT at this stage of the relay. Thus, the biathletes in G3 during legs 3 and 4 seem to have higher skiing capacity than G10, or they are outperforming as preliminarily suggested by Wolf et al. [18].

As shown in Fig. 1, the median ranks for different performance groups were comparable between women and men after legs 1, 2 and 3. However, after leg 2, G10 for women demonstrated larger range between minimum and maximum values compared to men. This is likely due to the longer RT observed for G10 during that leg, resulting in more separation among the women’s relay teams after leg 2. Conversely, for the G10 group in men, the teams remained more closely packed after leg 2.

It is notable for both women and men that although there were no statistically significant differences in many of these variables between G3 and G10, in terms of absolute values many non-significant differences may have practical importance. An additional note is that there were very small differences in the number of shots between groups; an indication that the shooting accuracy is relatively similar between groups. According to the present results, women and men combined, all three groups had a range of 7–11 extra shots (in relation to the 40 shots all teams need to fire, see Table 1) with a difference of only 2–3 shots between the groups, but with G3 having the lowest number of shots. This highlights, in general, the importance of high shooting accuracy together with short RT. In addition, re-loading and firing of one extra shot takes approximately 6–7 s, and thus G10, having two extra shots more than G3, accumulates approximately 12–14 s time loss on the shooting range in total, which would be difficult to re-gain during a relatively short skiing loop. In addition, G10 for women had almost 30 s longer RT, indicating that the teams in G10, especially biathletes on legs 2 and 4, also had a slower shooting performance. Conversely, G10 for men had only an eight seconds longer RT, regardless of two extra shots. This is partly in line with the previous biathlon studies regarding individual competition formats [4, 5] which have shown that women have longer range time compared to men. However, this must be considered with caution as also slower skiing speed for women when entering to the shooting range may affect the actual range time.

As biathlon combines both effort-based (i.e. skiing) and skill-based (i.e. shooting) performances, it is probably more challenging for coaches and team leaders to select biathletes for the team, as well as to decide the order of biathletes within the team compared to only effort-based sports (e.g. swimming, running). Previous studies on individual competition formats in biathlon, e.g [2, 4–6]., have highlighted the importance of shooting accuracy on final performance in biathlon. This is especially true when focusing on groups of biathletes at the highest level where the differences in skiing speed are particularly small, and in competition formats where the skiing distance is relatively short in comparison to the number of shooting occasions (such as pursuit and mass start). In biathlon relay, the total skiing distance is also short in relation to the number of shooting occasions, which therefore highlights the importance of shooting performance on final rank. Speculatively, if a biathlete misses two of the five ordinary shots, she/he needs to use at least two extra shots, which adds approximately 12–14 s against a biathlete who succeeds to shoot all five shots without any mistakes, assuming otherwise similar shooting times. If that biathlete still has a missed target after using extra shots, she/he is forced to ski a penalty loop, adding another 20–25 s, in total at least 32 s time loss. Thus, the relay is advantageous for biathletes with a high capacity for fast as well as accurate shooting without limiting capacity in skiing. This was especially shown with the present results regarding the women’s relay. Interestingly, individually better qualified teams (e.g. world cup points in individual competitions) will not necessarily perform better in relay, as has been suggested by Scharfenkamp et al. [19], who have also shown that increasing age diversity in the team significantly improves a team’s shooting performance in biathlon relay.

It needs to be mentioned that the present study did not consider the individual World Cup ranking of biathletes, i.e. comparisons of individual skiing, shooting, and overall performances within and between relay teams. This would be of interest for future studies, as speculatively, different nations may have different tactics for allocating biathletes to different legs (top-end team versus top-start team) to achieve the best possible final rank for the team and, for some teams, to avoid lapping during the competition. In addition, future studies should also consider analyzing the performance indicators in biathlon mixed relays, i.e. relay competitions including biathletes from both sexes.

## Conclusion

Taken altogether, the shooting performance for women (especially shorter RT and lower NS) during legs 2 and 4, and skiing performance for men during legs 3 and 4, are most decisive for final performance during a biathlon relay and discriminates the teams between ranks 1–3 (G3) and 4–10 (G10). Coaches should, therefore, consider these aspects when selecting biathletes into the relay team, as well as when planning their order within the team.

## Data Availability

The datasets used and/or analyzed during the current study are available from the corresponding author on reasonable request.

## Abbreviations

CT: Course time
G3: Group of rank 1–3
G10: Group of rank 4–10
G20: Group of rank 11–20
IBU: International Biathlon Union
IQR: Interquartile range
LT: Leg time
NPL: Number of penalty loops
NS: Number of shots
PT: Penalty time
RT: Range time
